# Facile Synthesis of Cobalt Ferrite (CoFe_2_O_4_) Nanoparticles in the Presence of Sodium Bis (2-ethyl-hexyl) Sulfosuccinate and Their Application in Dyes Removal from Single and Binary Aqueous Solutions

**DOI:** 10.3390/nano11113128

**Published:** 2021-11-19

**Authors:** Claudia Maria Simonescu, Alina Tătăruş, Daniela Cristina Culiţă, Nicolae Stănică, Bogdan Butoi, Andrei Kuncser

**Affiliations:** 1 Department of Analytical Chemistry and Environmental Engineering, Faculty of Applied Chemistry and Materials Science, Politehnica University of Bucharest, Polizu Street, No. 1-7, District 1, 011061 Bucharest, Romania; 2 National Research and Development Institute for Industrial Ecology, INCD-ECOIND, Drumul Podul Dambovitei Street, No. 71-73, District 6, 060652 Bucharest, Romania; 3 Ilie Murgulescu Institute of Physical Chemistry, 202 Splaiul Independentei, 060021 Bucharest, Romania; nstanica@icf.ro; 4 National Institute for Laser, Plasma and Radiation Physics, 077125 Măgurele, Romania; bogdan.butoi@inflpr.ro; 5 National Institute for Materials Physics, Atomistilor Street 405, 077125 Măgurele, Romania; andrei.kuncser@infim.ro

**Keywords:** anionic dyes adsorption, Congo Red, Methyl Orange, magnetic adsorbents, surfactant effects

## Abstract

A research study was conducted to establish the effect of the presence of sodium bis-2-ethyl-hexyl-sulfosuccinate (DOSS) surfactant on the size, shape, and magnetic properties of cobalt ferrite nanoparticles, and also on their ability to remove anionic dyes from synthetic aqueous solutions. The effect of the molar ratio cobalt ferrite to surfactant (1:0.1; 1:0.25 and 1:0.5) on the physicochemical properties of the prepared cobalt ferrite particles was evaluated using different characterization techniques, such as FT-IR spectroscopy, X-ray diffraction (XRD), scanning electron microscopy (SEM), transmission electron microscopy (TEM), N_2_ adsorption-desorption analysis, and magnetic measurements. The results revealed that the surfactant has a significant impact on the textural and magnetic properties of CoFe_2_O_4_. The capacity of the synthesized CoFe_2_O_4_ samples to remove two anionic dyes, Congo Red (CR) and Methyl Orange (MO), by adsorption from aqueous solutions and the factors affecting the adsorption process, such as contact time, concentration of dyes in the initial solution, pH of the media, and the presence of a competing agent were investigated in batch experiments. Desorption experiments were performed to demonstrate the reusability of the adsorbents.

## 1. Introduction

With the rapid growth of industrialization and the increase of human needs, the global production of products that require coloring and the volume of wastewater containing dyes have increased. Dyes are colored natural or synthetic organic substances that absorb light in the visible range of the spectrum and have the property of giving color to other substances or materials. More than 100,000 dyes with an annual production of over 7 × 105 tones/year are commercially available [1]. Two thirds of the dyes are used in the textile industry [2] and an important volume of dye-containing wastewater is released into the environment. The negative effects of dyes are observed in natural ecosystems and on human health. Dyes are characterized by high optical and thermal stability, which makes their persistence in the environment to be ongoing for a long time. In aquatic ecosystems, dyes consume the dissolved oxygen, absorb and reflect the light and negatively influence the photosynthesis and growth of bacteria, as well as the aquatic life and food chain in these ecosystems. Many of the dyes are toxic, mutagenic, and carcinogenic and cause allergies in the organisms. The chronic and/or acute effects of dyes on exposed living organisms depend on the dye level and on the exposure time. As a result, the removal and recovery of dyes from water is a priority, as even low concentrations can cause serious damage to living organisms and the environment. Congo Red (CR) and Methyl Orange (MO) are two synthetic anionic azo dyes highly soluble in water and ethanol that are persistent and difficult to biodegrade after their release into the environment. The low biodegradability of CR and MO is due to their complex molecular structure and the presence of the azo group (N=N). They are widely used in the textile, printing, wood, paper, leather, plastic, pharmaceutical and food processing industries for diagnosis purposes, and as pH indicators in research laboratories [3,4].

Traditional and non-conventional measures and methods are involved in dye removal from water and wastewater. As traditional methods widely applied to remove CR and MO from water, aqueous solutions and wastewater can be mentioned: coagulation and flocculation [5,6], adsorption [7,8], advanced oxidation techniques [9], electrochemical oxidation [10], photo Fenton process [11], photocatalytic degradation [12], and membrane-based processes [13,14]. Unconventional methods used to remove CR and MO for aqueous effluents are: biosorption [15,16], bioremediation [17], biodegradation [18], microwave-enforced sorption processes [19], sonocatalytic degradation [20], and ultrasound-assisted adsorption [8]. Numerous adsorbents such as: activated carbon and its composites [21,22], biochar [23,24], layered double hydroxide (LDH) [7,8], metallic oxides and their composites [25,26], calcium aluminate hydrates [27], zeolites and their composites [6], clays [28,29], magnetic materials [24,30], hydroxyapatite and its derivatives [31,32], synthetic polymeric materials [6,33], natural polymers [34,35], metal-organic frameworks [14,36], and industrial waste [37,38] have been applied for CR and MO removal. Magnetic materials are an important class of adsorbent materials used to remove both organic and inorganic pollutants from wastewater. The main advantage of these types of materials is the ease of their separation from the treated water after the process of retaining pollutants by a simple application of an external magnetic field. Consequently, numerous studies have been devoted to assessing the use of diverse types of magnetic nanoparticles, such as ferrites (MFe_2_O_4_, M=Co, Ni, Mn, Fe, Mg, Ca, Zn) and their functionalized derivatives for dye removal and recovery [39,40,41,42,43,44,45,46]. The interesting properties of cobalt ferrite, such as physical/chemical stability, mechanical hardness, and the possibility to be easily separated/recovered from the treated water by applying a magnetic field, have led to numerous studies on the use of this material in the retention of pollutants in wastewater [39]. The main synthetic methods of cobalt ferrite are: co-precipitation, combustion, sol-gel, solid state, hydrothermal, micro-emulsion, mechanical milling, reverse micelles, spray-drying, and ultrasonic and microwave-assisted hydrothermal processes [39]. The method and the synthesis parameters have an impact on the morphological, structural, textural, and magnetic properties of cobalt ferrite. Furthermore, the experimental conditions can be modified for improving and controlling the cobalt ferrite properties. Hydrothermal synthesis methods in the presence of surfactants are of great interest due to their low cost and simplicity, but also due to the control of the size, shape, and crystalline phases of the cobalt ferrite nanoparticles. The effect of surfactants such as cetyltrimethylammonium bromide (CTAB) [47,48], oleylamine, and a mixture of oleic acid/oleylamine [49], di iso-octyl sulphosuccinate [50], oleic acid [51], and sodium dodecyl sulfate (SDS) [52] on cobalt ferrite properties have been investigated.

The objective of the present study is to explore the adsorption potential of cobalt ferrite nanoparticles prepared by a surfactant (sodium bis-2-ethyl-hexyl sulfosuccinate (DOSS))-assisted hydrothermal synthesis method. The effect of DOSS on cobalt ferrite properties such as specific surface (S_BET_), magnetic properties and dye adsorption capacity was investigated. Two synthetic anionic azo dyes such as Congo Red (CR) and Methyl Orange (MO) were used as model dyes for adsorption experiments in single and binary aqueous synthetic solutions. The effect of experimental conditions, kinetic assessment, adsorption mechanism, and adsorption isotherms was also investigated.

## 2. Materials and Methods

### 2.1. Materials

The cobalt ferrite samples were prepared by the use of Fe(NO_3_)_3_·9H_2_O 99%, Co(NO_3_)_2_·6H_2_O and sodium bis-2-ethyl-hexyl sulfosuccinate (analytical grade, Sigma Aldrich, St. Louis, MO, USA). NH_4_OH 25% and HCl 35% suprapure (analytical grade, Merck KGaA Germany, Darmstadt, Germany) were used to adjust the solution’s pH. The dye solutions of desired concentrations were obtained from Congo Red (CR) 99% (C_32_H_22_N_6_Na_2_O_6_S_2_) and Methyl Orange (MO) (ACS reagent, Dye content 85%, C_14_H_14_N_3_NaO_3_S, Sigma Aldrich). Ethyl alcohol (95%) (Sigma Aldrich, St. Louis, MO, USA) was applied as a desorbing agent. Ammonium acetate 99% and acetonitrile 99% (Merck KGaA Germany, Darmstadt, Germany) were utilized as a mobile phase for the dye’s chromatographic separation and detection.

### 2.2. Characterization Methods and Equipment

The analytical techniques, such as Fourier transform infra-red (FT-IR) analysis, X-ray diffraction (XRD), N_2_ sorption analysis, scanning electron microscopy (SEM)/transmission electron microscopy (TEM), and magnetic measurements have been involved in cobalt ferrite sample characterization.

FT/IR spectra (4000–400 cm^−1^) were recorded on a JASCO FT/IR-4700 spectrometer (Tokyo, Japan) by the use of KBr pellets. The structural characterization (XRD analysis) was performed with a Bruker A8 Advanced diffractometer in a Bragg-Brentano configuration, equipped with a 1D LynxEye detector and an X-ray-emitting tube with a copper anode. The recording of X-ray diffractograms was performed in the range 2θ (6 ÷ 85°), with a step of 0.02° and a cumulative acquisition time of 118 s. The incident divergent beam was collimated with a 0.6 mm slit, and the K_β_ radiation was removed with a nickel filter leaving only the CuK_α1, 2_ radiation (λ_medium_ = 0.154178 nm). The calculations regarding the average crystallite size and the identification of the crystalline phases were performed using Scherrer’s formula and the ICDD (International Center of Diffraction Data) database, respectively. The specific surface areas (S_BET_) were determined from N_2_ adsorption-desorption experiments at −196 °C performed on a Micromeritics ASAP 2020 automatic adsorption system (Norcross, GA, USA). The samples were degassed at 250 °C for 5 h under vacuum before analysis. SEM images were recorded on an FEI Inspect S microscope (Hillsboro, OR, USA). The samples were investigated in a high vacuum medium (<10–3 mbar), at a working distance of 10 mm, with an acceleration voltage of 25 kV, ~60 uA current). Transmission Electron Microscopy (TEM) has been involved in the morphological and structural characterization of the CoFe_2_O_4_ samples. A JEOL 2100 instrument, equipped with a LaB6 electron gun and high resolution polar piece has been used. Magnetic properties were assessed at room temperature on Lake Shore’s fully integrated Vibrating Sample Magnetometer system 7404 (VSM) (Westerville, OH, USA). Batch tests were carried out at 150 rpm (rotation per minute) on a GFL 3015 orbital shaker (Burgwedel, Germany) to evaluate the cobalt ferrite adsorption capacity. The dye solution’s pH was measured at room temperature on an Agilent 3200 laboratory pH-meter (Agilent Technologies, Shanghai, China).

**Figure 1:**
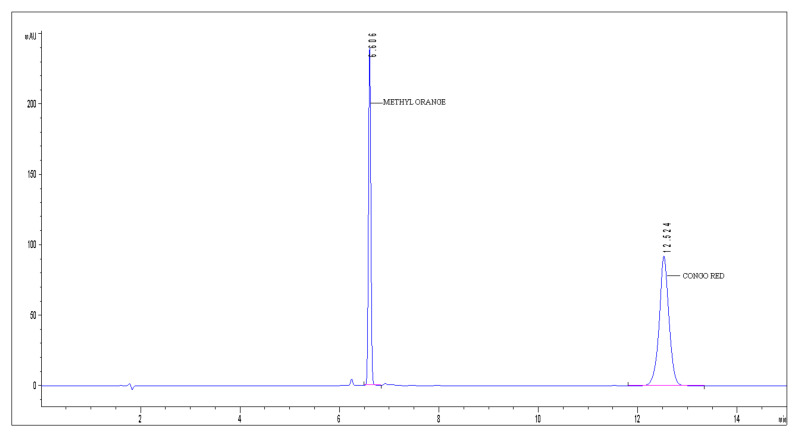
The chromatogram obtained from the analysis of a mixed solution of MO and CR.

The dye’s chromatographic separation and detection were achieved on an Agilent 1200 series HPLC (Tokyo, Japan) equipped with a Diode Array Detector (DAD) that records simultaneously UV-VIS spectra (190–900 nm). The Acclaim Surfactant Plus column (150 × 3.0 mm, 3.0 µm) (Thermo Scientific, Tokyo, Japan) was utilized for chromatographic runs. The detection of dye was performed at the optimal wavelengths: λ = 506 nm (CR) and λ = 428 nm (MO). Data acquisition, processing, and reporting was carried out by the use of Agilent ChemStation software, Santa Clara, CA, USA (version B03.02). The development of the high-performance liquid chromatographic method with diode array detector (HPLC-DAD) and experimental conditions and results for detection of CR/MO from synthetic aqueous solution are already presented in our previous works [53,54]. The chromatogram obtained from the analysis of a mixed solution of MO and CR is shown in Figure 1. The retention time for MO was approximately 6 min, while for Congo Red it was approximately 12 min.

### 2.3. Synthesis Protocol

For the synthesis of the CoFe_2_O_4_ samples, 4 experiments were performed. In the first experiment, CoFe_2_O_4_ was synthesized by coprecipitation from solution. Briefly, a solution of Fe(NO_3_)_3_·9H_2_O (8.08 g dissolved in 100 mL distilled water) was mixed with a solution of Co(NO_3_)_2_·6H_2_O (2.91 g dissolved in 50 mL distilled water). After stirring for 30 min at room temperature, 80 mL of 25% NH_4_OH was added and the mixture was further stirred for another 2 h at 80 °C. After cooling, the precipitate was filtered and washed with distilled water and ethyl alcohol. The black powder obtained was dried for 4 h at 80 °C in an oven.

**Figure 2:**
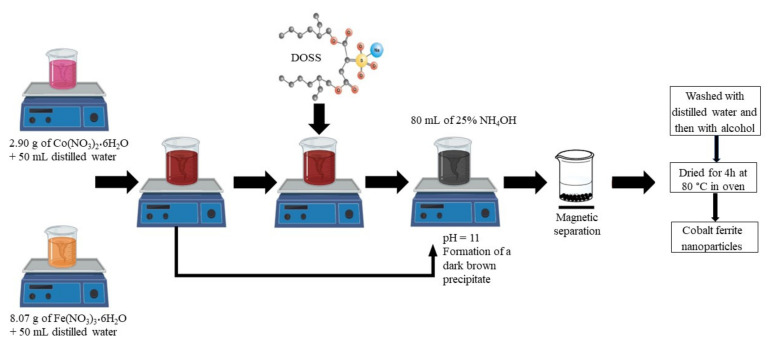
Synthesis of cobalt ferrite in absence and in presence of surfactant (DOSS).

The other 3 experiments were performed using different molar ratios of cobalt ferrite to surfactant (1:0.1; 1:0.25 and 1:0.5). The surfactant was added to the reaction mixture before changing the pH (Figure 2).

Cobalt ferrite samples were denoted as follows: CoFe_2_O_4_-0—sample synthesized in the absence of surfactant, CoFe_2_O_4_-0.1, CoFe_2_O_4_-0.25, and CoFe_2_O_4_-0.5—samples synthesized in the presence of the surfactant using molar ratios of CoFe_2_O_4_:surfactant = 1:0.1, 1:0.25, and 1:0.5.

### 2.4. The Adsorption and Desorption Test

The effect of the experimental parameters was established in batch experiments at room temperature (22 ± 2 °C). A volume of 15 mL of azo dye (CR/MO/CR + MO) of an initial concentration of 100 mg·L^−1^ was contacted with 0.005 g of cobalt ferrite for 10 to 240 min at 150 rpm. After the set period of time, the adsorbent (cobalt ferrite) was separated by applying a magnetic field, and the residual concentration of the dyes in solution was determined by HPLC.

The experiments regarding the effect of the dye solution’s pH were performed in the range of pH between 2 and 11. An isothermal study was conducted in the dye concentration range from 5 to 100 mg·L^−1^. Single and binary dye solutions were analyzed. Six recyclability tests were performed by contacting 15 mL of an elution agent with 0.005 g of dye-loaded cobalt ferrite for 4 h, at room temperature and 150 rpm. The amount of CR/MO retained per gram of CoFe_2_O_4_, the desorption efficiency, and the desorption capacity are calculated with the following equations:

(1) $$Q_{t}=\frac{\left(C_{0}-C_{t}\right)V}{m}$$

where: Q_t_ is the removal capacity defined as the amount of CR/MO retained per gram of CoFe_2_O_4_ at various contact times (mg·g^−1^), C_0_ means the CR/MO initial concentration (mg·L^−1^), C_t_ defines the CR/MO concentration after time (t) of contact with CoFe_2_O_4_ (or at various pH values) (mg·L^−1^), V represents the CR/MO solution volume (L), and m defines the amount of CoFe_2_O_4_ (g).

(2) $$D\left(\%\right)=\left(\frac{Q_{D}}{Q_{e}}\right)\times 100$$

where: D represents the desorption efficiency (%), Q_D_ means the desorption capacity estimated by Equation (3) (mg·g^−1^) and Qe is the adsorption capacity at equilibrium (mg·g^−1^).

(3) $$Q_{D}=\frac{C_{f}}{m}\times V$$

where: C_f_ is the final concentration of CR/MO desorbed (mg·L^−1^), V means the eluent agent volume (L), and m constitutes the amount of CoFe_2_O_4_ loaded with CR/MO (g).

The batch tests were carried out in triplicate with a maximum experimental error of 5%.

## 3. Results and Discussion

### 3.1. Spectroscopic Characterization of CoFe_2_O_4_ Samples (FTIR Spectroscopy)

**Figure 3:**
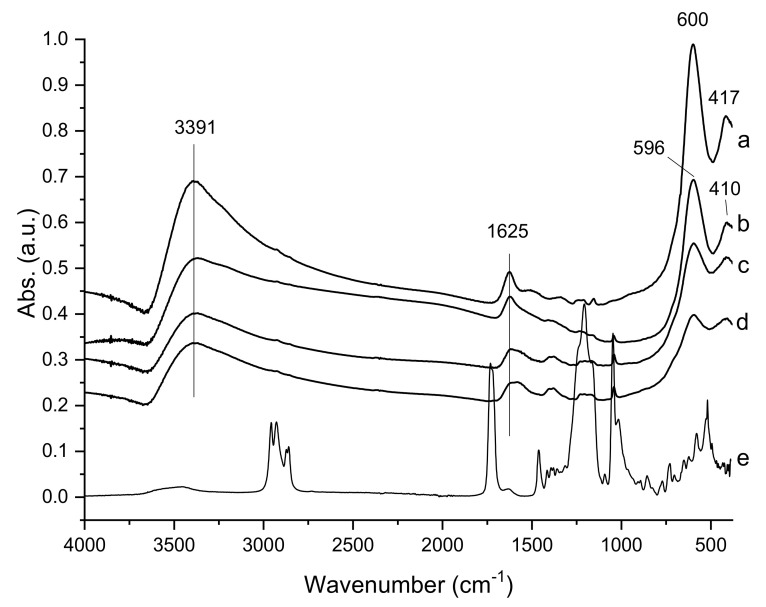
FTIR spectra of (a) CoFe_2_O_4_—0; (b) CoFe_2_O_4_—0.1; (c) CoFe_2_O_4_—0.25; (d) CoFe_2_O_4_—0.5; (e) DOSS.

The FTIR spectra of CoFe_2_O_4_, DOSS surface-modified CoFe_2_O4 samples, and free DOSS recorded in the range of 4000–400 cm^−1^, are shown in Figure 3.

The spectrum of CoFe_2_O_4_-0 reveals the presence of two intense absorption bands at 600 and 417 cm^−1^ which are associated with tetrahedral–A and octahedral–B sublattices of pure CoFe_2_O_4_ particles, according to the literature data [55,56]. These absorption bands confirm the presence of Co-O and Fe-O bonds in the cobalt ferrite structure. These two bands are slightly shifted to lower frequencies, 596 and 410 cm^−1^ in the spectra of the DOSS surface-modified CoFe_2_O_4_ samples. This shifting can be attributed to the changes in the length of the M-O bonds in the tetrahedral and octahedral sites [55]. The bands located at 3391 cm^−1^ and 1625 cm^−1^ are characteristic of the O–H stretching vibration and H–O–H bending vibration [55]. This indicates the presence of hydroxyl groups and water molecules absorbed on the surface of the CoFe_2_O_4_ particles. By comparing the spectra of DOSS surface-modified CoFe_2_O_4_ samples with that of free DOSS, it is clear that the other bands present in the DOSS surface-modified CoFe_2_O_4_ samples in the range of 1000–1500 cm^−1^ could be attributed to the surfactant traces entrapped in the small pores.

### 3.2. Structural and Morphological Characterization of CoFe_2_O_4_ Samples

**Figure 4:**
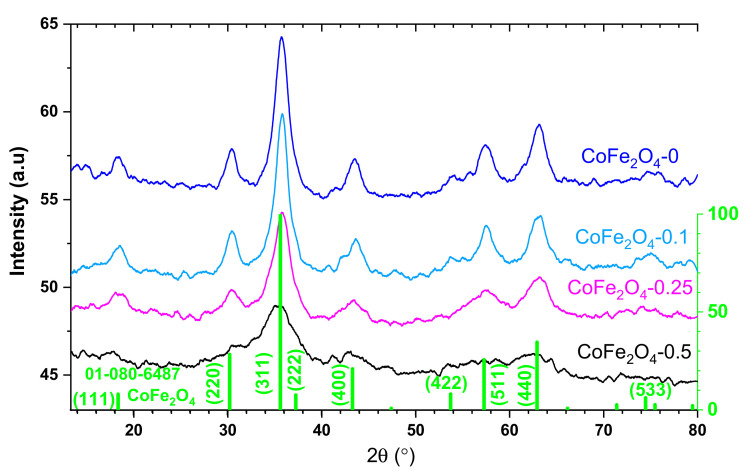
X-Ray diffractograms of CoFe_2_O_4_ samples.

X-ray diffractograms for each powder are shown in Figure 4. They are similar (therefore the same polycrystalline phases are present in all CoFe_2_O_4_ powders), the differences from one sample to another being given by the position and width of the diffraction maxima (therefore, the dimensions of the elementary cell and the average size of the crystallites differ). All diffraction maxima indicate that there is a major (98%) cubic phase of the spinelic type, CoFe_2_O_4_, according to the PDF sheet 01-080-6487, which is represented in Figure 4 by the green vertical bars. Most probably the difference of 2% could be attributed to some secondary phases such as iron or cobalt oxides.

The lattice parameter a, and the mean size (D) of the CoFe_2_O_4_ crystallites were calculated for each powder from the parameters of the maximum (311). The results are presented in Table 1. According to these values, the CoFe_2_O_4_ was synthetized as nanoparticles and the effect of the surfactant consisted of a small decrease of the particle size.

**Figure 5:**
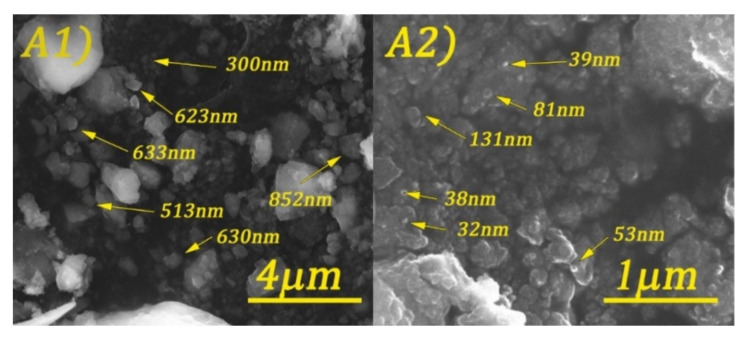
SEM images of CoFe_2_O_4_-0 at different magnifications: 20 kX (**A1**) and 80 kX (**A2**).

**Figure 6:**
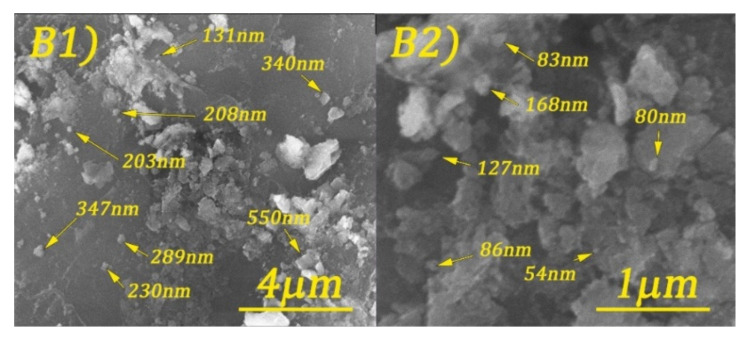
SEM images of CoFe_2_O_4_-0.1 at different magnifications: 20 kX (**B1**) and 80 kX (**B2**).

**Figure 7:**
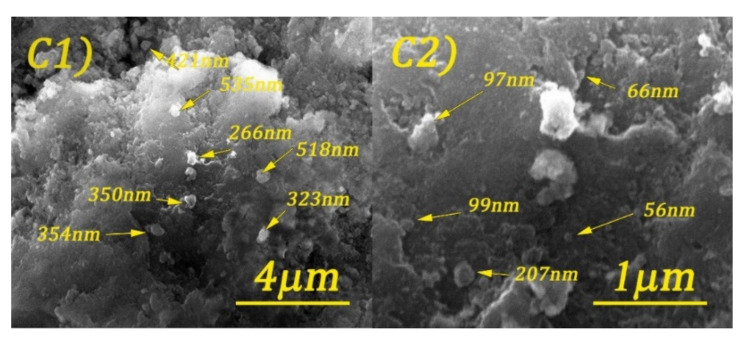
SEM images of CoFe_2_O_4_-0.25 at different magnifications: 20 kX (**C1**) and 80 kX (**C2**).

**Figure 8:**
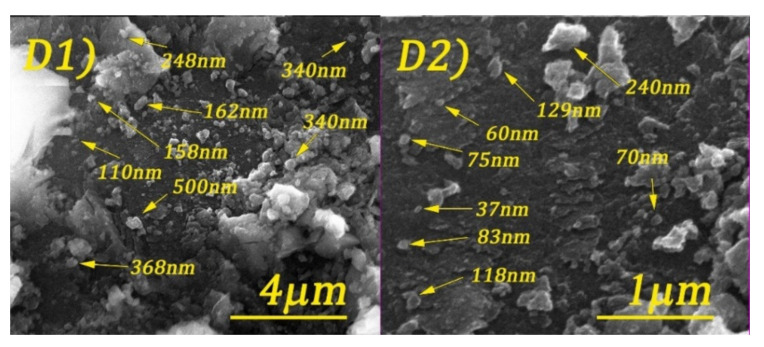
SEM images of CoFe_2_O_4_-0.5 at different magnifications: 20 kX (**D1**) and 80 kX (**D2**).

Scanning electron microscopy (SEM) images of cobalt ferrite and DOSS surface-modified cobalt ferrite are shown in Figure 5, Figure 6, Figure 7 and Figure 8.

By analyzing the above figures, it can be seen that the CoFe_2_O_4_ was prepared in the form of conglomerates of nanometric and micrometric structures. Particle sizes range from tens of nanometers to micrometers. The effect of the surfactant is observed by the formation of much better-defined particles, but with less regular shapes.

**Figure 9:**
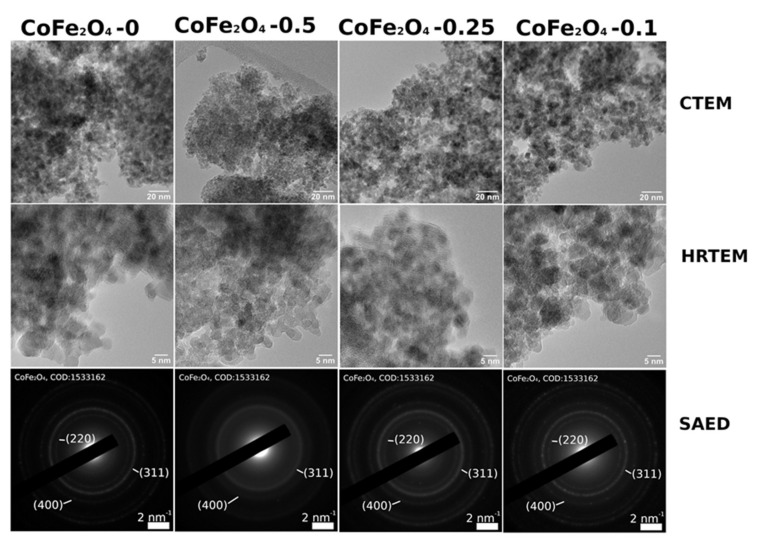
A comparative approach of CTEM images, HRTEM images and SAED profiles as obtained on CoFe_2_O_4_ samples.

Conventional Electron Microscopy (CTEM) images, High Resolution Transmission Electron Microscopy (HRTEM) images, and Selected Area Electron Diffraction (SAED) profiles have been obtained and are shown in Figure 9.

Statistics on nanoparticles (NPs) sizes as obtained via TEM imaging are as follows: 5.46 ± 1.54 nm for CoFe_2_O_4_-0, 5.19 ± 1.77 nm for CoFe_2_O_4_-0.1, 4.12 ± 1.03 nm for CoFe_2_O_4_-0.25, and 3.77 ± 0.64 nm for CoFe_2_O_4_-0.5. Although all samples have a similar morphology, i.e., small-faceted nanoparticles (NPs) with a general quasi-spherical aspect, with diameters roughly in the 3–6 nm range, there are some differences worth mentioning. CoFe_2_O_4_-0, CoFe_2_O_4_-0.1, and CoFe_2_O_4_-0.25 show well-crystallized, faceted nanoparticles with a mean NP size of 5–6 nm, whereas the NPs in the CoFe_2_O_4_-0.5 sample appear noticeably smaller, with a more pronounced spherical-like aspect, with an average diameter of 3–4 nm. According to SAED patterns, while all the samples present NPs crystallized in the CoFe_2_O_4_ phase, the crystallinity of the CoFe_2_O_4_-0.5 system is lower than in all other cases.

### 3.3. Textural Properties of CoFe_2_O_4_ Samples

**Figure 10:**
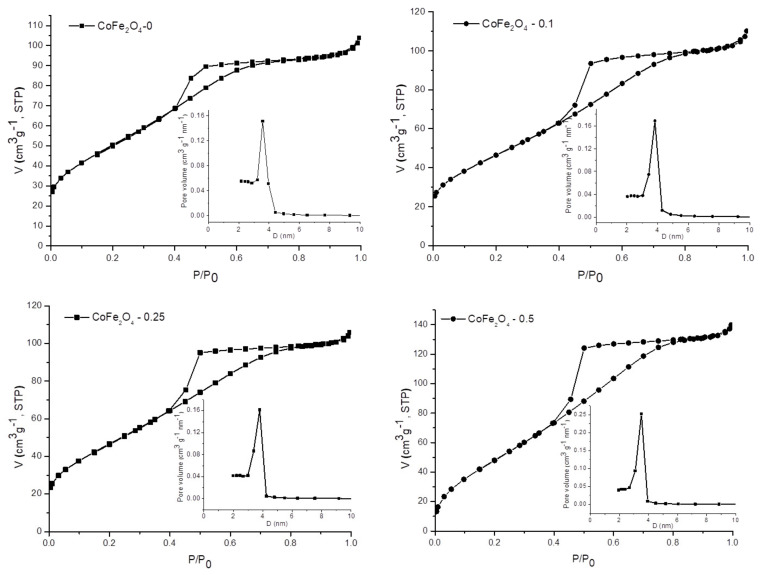
N_2_ adsorption−desorption isotherms and pore size distribution (inset) of the samples.

The textural properties of the samples were investigated by nitrogen adsorption-desorption analysis. Figure 10 shows the N_2_ sorption isotherms and the corresponding pore size distribution curves. The values of specific surface areas, total pore volumes, and average pore diameters are listed in Table 1. All isotherms are type IV according to the IUPAC classification with hysteresis loops of H2 type, which is associated with capillary condensation phenomena in mesoporous structures. The H2-type hysteresis loops indicate the presence of complex pore networks. The pore-size distribution of each sample was calculated from the desorption branch of the isotherms using the BJH model. A monomodal and narrow distribution of pore sizes for all four samples can be observed, with close average diameters ranging from 3.3 to 3.7 nm. In addition, it can be seen that the average pore diameter slightly decreases as the amount of surfactant increases. We can assume that this behavior could be due to traces of surfactant entrapped inside the pores, as evidenced by FTIR spectra (Figure 3). Considering the intensity of the bands assigned to the surfactant in the FTIR spectra of the samples, we can say that the amount of surfactant entrapped inside the pores is directly proportional to the amount of surfactant used in the synthesis. The sample CoFe_2_O_4_–0.5 that was obtained using the largest amount of surfactant has the highest BET surface area and total pore volume, as expected, while CoFe_2_O_4_–0.1 and CoFe_2_O_4_–0.25 have lower values, but close to each other, for these two parameters. However, the surface areas and total pore volumes of all four samples are higher than those reported in other papers in which the surfactant-assisted method has been used [47].

### 3.4. Magnetic Characterization of CoFe_2_O_4_ Samples

**Figure 11:**
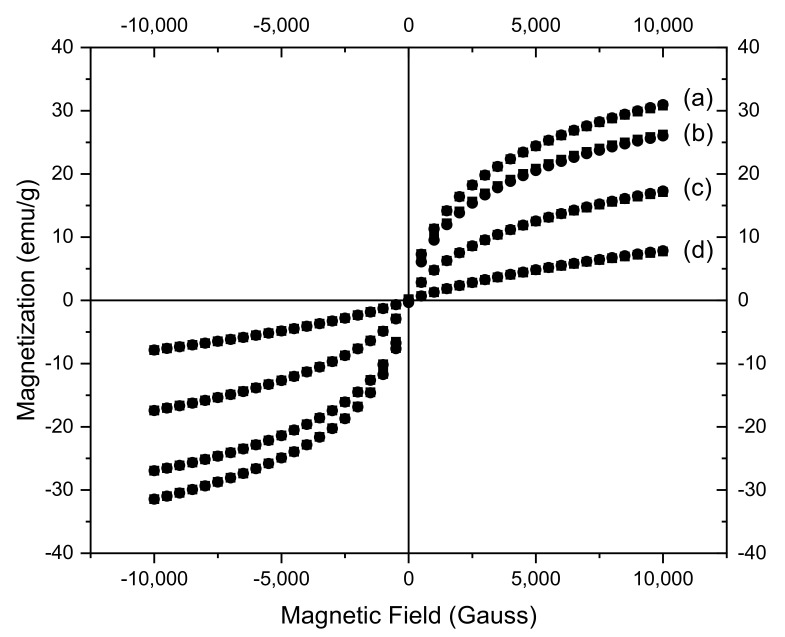
Magnetization curves of (a) CoFe_2_O_4_−0; (b) CoFe_2_O_4_−0.1; (c) CoFe_2_O_4_−0.25; (d) CoFe_2_O_4_−0.5 at room temperature.

Magnetic properties of the samples were measured by VSM. Figure 11 shows the magnetization versus magnetic field curves at room temperature. The zero values of remanence magnetization and coercivity observed on the hysteresis curves indicate that all CoFe_2_O_4_ samples are superparamagnetic. The calculated saturation magnetization (M_s_) values (Table 1) are smaller than that reported for bulk CoFe_2_O_4_ which is 72 emu/g [57]. This can be due to the smaller size of the nanoparticles and surface effects [58]. It can be noted that the decrease of the Ms values is proportional with the amount of surfactant used in the synthesis.

### 3.5. Removal of Anionic Dyes from Single and Binary Solutions by Adsorption on CoFe_2_O_4_ Samples

Magnetic materials are important in the field of pollutants removal from wastewater due to their high adsorption capacity, but also to the magnetic properties which make them suitable for the separation processes. In this work, the removal of two anionic dyes, Congo Red and Methyl Orange, by adsorption on cobalt ferrite prepared in the absence/presence of surfactant was investigated in batch experiments. The effect of some important parameters on the removal capacity of these materials was studied in order to establish the influence of the amount of surfactant on their adsorption capacity and to determine the optimal conditions of the dye-removal process. Among the parameters investigated that can be mentioned are: the contact time, the solution’s pH, the concentration of dyes in the initial solution, and the presence of a competing agent. To investigate the last parameter, the experiments were performed using binary solutions of Congo Red and Methyl Orange.

#### 3.5.1. The Effect of the Contact Time and Fitting of Kinetic Models to the Adsorption Data

**Figure 12:**
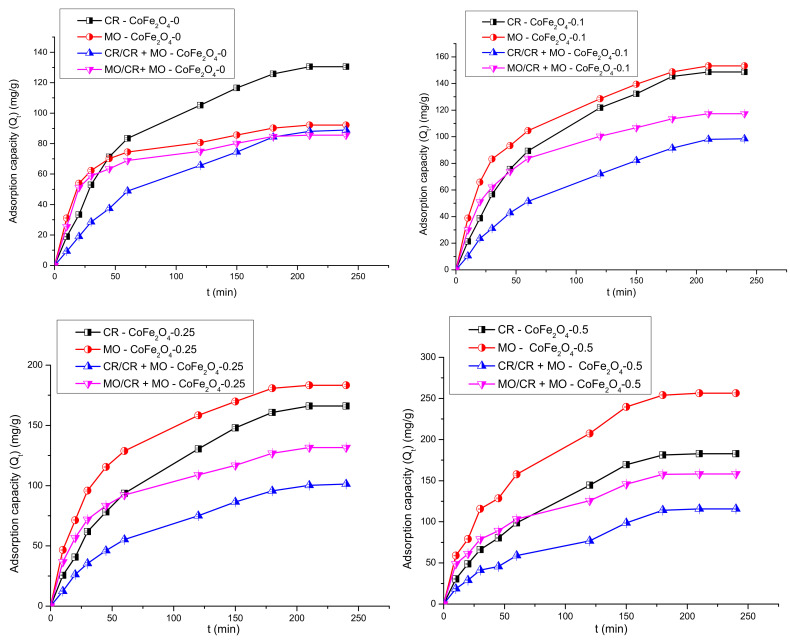
Effect of contact time on the adsorption capacity of CoFe_2_O_4_−0, CoFe_2_O_4_−0.1, CoFe_2_O_4_−0.25, and CoFe_2_O_4_−0.5 from single and binary solutions.

The effect of contact time on the adsorption capacity of the CoFe_2_O_4_ samples was studied in single and binary solutions and the results were discussed comparatively. The variation of the adsorption capacity of the CoFe_2_O_4_ samples for CR/MO from single and binary aqueous solutions as a function of the contact time is illustrated in Figure 12.

Analyzing the data, it can be observed that the adsorption capacity of the cobalt ferrite samples increases with contact time. Initially, the speed of the retention process is high due to the abundance of free active centers on the surface of the cobalt ferrite. By increasing the contact time, the number of free active centers decreases, and consequently, the retention process slows down. In single solutions, a higher retention capacity for MO compared to CR for all CoFe_2_O_4_ samples synthesized in the presence of the surfactant was observed, with the highest values recorded for CoFe_2_O_4_-0.5 (MO—256.3 mg·g^−1^, CR—182.7 mg·g^−1^). It was also found that the adsorption capacity increases with the increase of the molar ratio cobalt ferrite: surfactant. In the case of MO adsorption from binary solutions (CR + MO) a decrease of the adsorption capacity compared to that obtained for single solutions was observed. It can be noted that for CoFe_2_O_4_-0 the decrease of the adsorption capacity for MO in binary solutions compared to single solutions was of 6.65 mg·g^−1^, while for CR the decrease was higher, 41.64 mg·g^−1^. For all CoFe_2_O_4_ samples synthesized in the presence of the surfactant, the decrease of the adsorption capacity was almost similar for both dyes. Based on these experiments, the contact time of 4 h was selected for further investigation.

A kinetic study was performed to determine the type of interaction between the active centers of the adsorbent and the dyes. Experimental data were evaluated by three kinetic models that are most often used to determine the mechanism involved in the adsorption process: the pseudo-first-order kinetic that is indicative for the domination of physisorption, the pseudo-second-order kinetic model that is based on the assumption that the adsorption process takes place through chemical reactions between the active centers of the adsorbent and adsorbate, and the intraparticle diffusion model which presumes that the interaction between the pollutant and active sites of the adsorbent is instantaneous relative to diffusion steps and consequently, these diffusion steps control the overall rate [59]. The equations of the three kinetic models are shown in Table 2.

**Figure 13:**
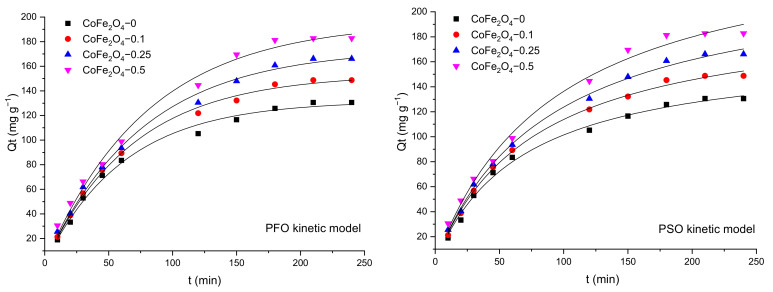
Graphical representation of the PFO and PSO kinetic models for removal of CR by adsorption onto CoFe_2_O_4_ samples from single solutions (nonlinear regression).

**Figure 14:**
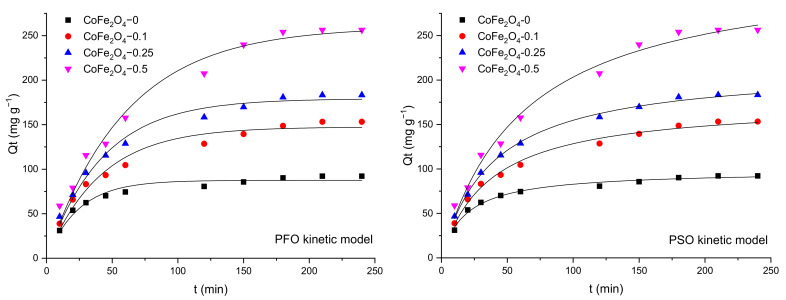
Graphical representation of the PFO and PSO kinetic models for removal of MO by adsorption onto CoFe_2_O_4_ samples from single solutions (nonlinear regression).

**Figure 15:**
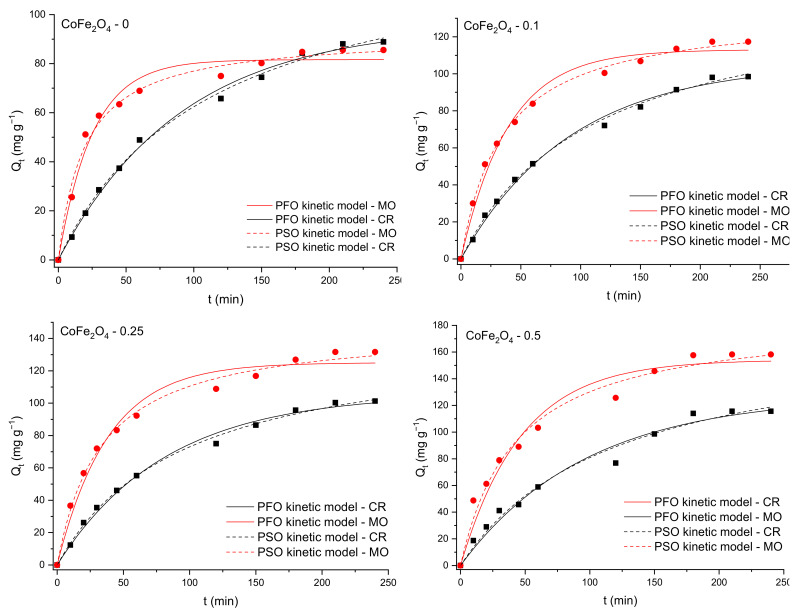
Graphical representation of the PFO and PSO kinetic models for removal of CR and MO by adsorption onto CoFe_2_O_4_ samples from binary solutions (nonlinear regression).

Figure 13, Figure 14 and Figure 15 show the pseudo-first order (PFO) and pseudo-second order (PSO) kinetic models fitting the adsorption data for CR and MO in single-component and binary solutions. Table 3 and Table 4 display the values of the kinetic parameters calculated using the PFO and PSO nonlinear models in single component and binary solutions, respectively. In the case of CR adsorption, the values of the adjusted R^2^ (>0.99) are almost similar for the PFO and PSO models. Considering the consistencies between the experimental and calculated Q_e_ values, it seems that the PFO model is more applicable for the removal kinetics of the CoFe_2_O_4_ samples toward the dye. In the case of MO adsorption, the values of the adjusted R^2^ (>0.99) are slightly higher for the PSO model than for the PFO model, but the calculated Q_e_ values from the PSO kinetic model do not agree well with the experimental data. For this reason, it can be considered that the PFO kinetic model is more feasible to describe the adsorption process of MO onto the CoFe_2_O_4_ samples.

**Figure 16:**
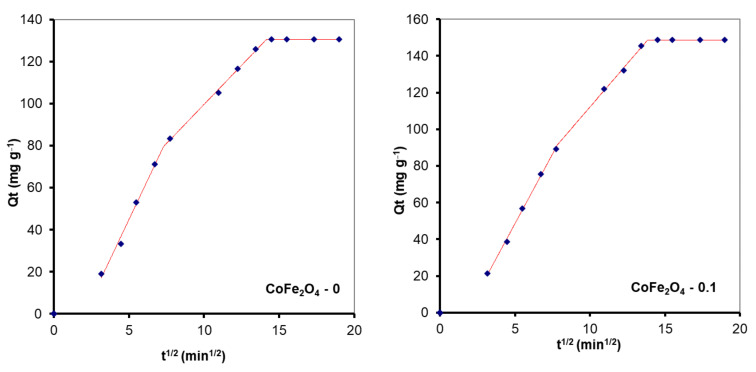

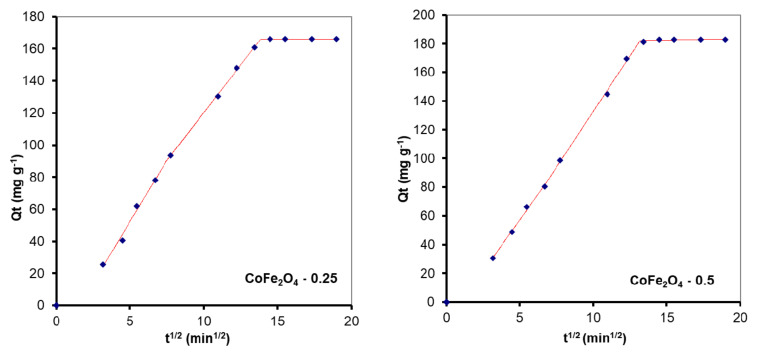
Kinetic modelling of the experimental data obtained from the sorption process of CR onto CoFe_2_O_4_ samples using the intraparticle diffusion model.

**Figure 17:**
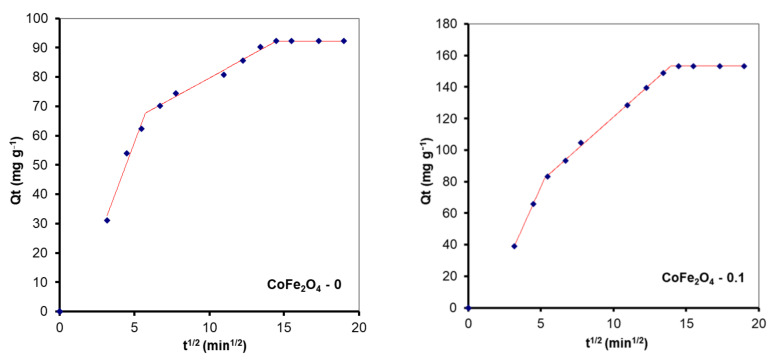

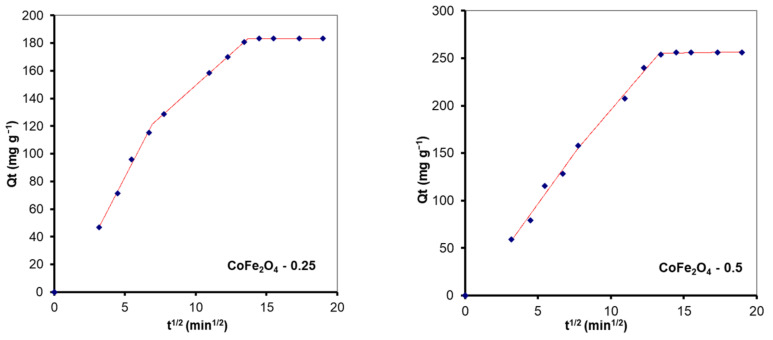
Kinetic modelling of the experimental data obtained from the sorption process of MO onto CoFe_2_O_4_ samples using the intraparticle diffusion model.

According to some studies, external mass transfer occurs at the beginning of the adsorption process, followed by intraparticle diffusion [60]. In the first stage, there is a high concentration gradient between the aqueous and adsorbent surfaces, which causes the solute to migrate faster onto the adsorbent surface. After a period of time, intraparticle diffusion to the adsorbent’s internal adsorption sites occurs, which is a sluggish process. This behavior is described by the Weber-Morris model, also known as the intraparticle diffusion model (Equation (6)). According to this model, if a straight line passing through the origin is generated from the plot of Equation (6), it can be inferred that the adsorption mechanism involves intraparticle diffusion of the species. The slope of the linear curve is the rate constant of the intraparticle diffusion process [61]. However, in the current investigation the plot is not linear and the trend line does not pass through the origin, indicating that intraparticle diffusion is not the only rate-limiting step in dye adsorption on CoFe_2_O_4_ samples. A piecewise linear regression was applied to the experimental data using a Microsoft Excel worksheet developed by Malash and El-Khaiary [62]. The results are presented in Figure 15 and Figure 16 and Table 5. As seen in Figure 16 and Figure 17, the adsorption process can be described based on three stages represented by three straight lines. The first one, characterized by a steep slope, indicates rapid adsorption of the dye at the external surface of the adsorbent. The second stage, in which the slope gradually becomes less slanting, is mainly associated with the diffusion of dye molecules through the internal pores of the adsorbent. The third line is almost horizontal in the last stage, indicating that the equilibrium condition has been reached. This pattern suggests that adsorption is controlled by both external mass transfer and intraparticle diffusion.

#### 3.5.2. The Effect of pH on the Adsorption Performance

**Figure 18:**
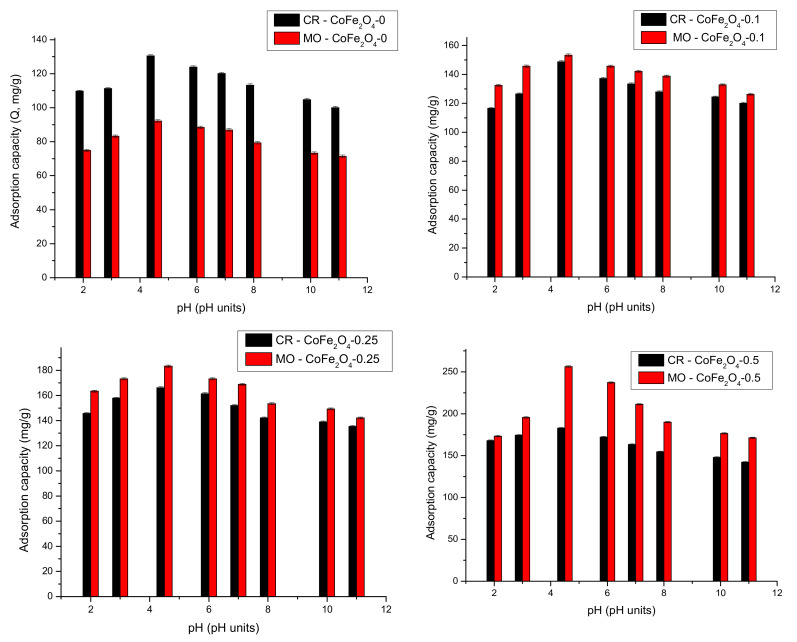
Effect of pH on adsorption capacity of CoFe_2_O_4_ samples for CR/MO from single solutions.

The effectiveness and selectivity of a wastewater remediation process by adsorption are influenced by the pH value. It can influence the surface electric charge of the adsorbent and adsorbate and consequently, the nature of the interactions involved in the removal mechanism. The tests regarding the effect of pH on the removal performance of the studied magnetic materials were conducted in the pH range 2–11 and the results are displayed in Figure 18.

It can be observed that the adsorption capacity of all cobalt ferrite samples increases with the increasing of the solution pH up to 4.5. After this pH value, the adsorption capacity decreases with the increasing of the solution’s pH up to 11. This can be explained by the fact that at a lower pH, the positively-charged surface of the CoFe_2_O_4_ samples interacts electrostatically with the negative sulfonate groups of CR/MO. The highest adsorption capacity of the CoFe_2_O_4_ samples was experimentally established to be at a pH of approximately 4.5. At a higher pH, the concentration of OH^-^ ions continues to increase, competing with the dye molecules for the adsorption sites. Repulsive forces between the dyes and the adsorbent are stronger in basic conditions, hence the adsorption capacity decreases with the increasing of pH values. The results are relatively close to the findings reported by Zwane et al. [13] and Simonescu et al. [54]. The same behavior was found for dye removal from binary solutions. Therefore, the pH value of 4.5 was considered optimal and was used in subsequent experiments.

#### 3.5.3. The Adsorption Isotherms

Adsorption isotherms provide information about the interactions established between the adsorbent and adsorbate during the adsorption process, the mechanism of adsorption, and the efficiency of the adsorbent in terms of its potential to be applied at laboratory, pilot, and industrial scale. Adsorption isotherms describe the relationship between the concentration of the solute retained on the adsorbent surface and its concentration in solution, under well-defined experimental conditions (at optimal contact time and pH, a certain temperature). These values are most often determined experimentally.

Regardless of whether they are inorganic or organic pollutants, the adsorption process can be characterized using the Langmuir and Freundlich isotherms. The nonlinear forms of equations that describe these isotherms are presented in Table 6.

In accordance with the Langmuir isotherm model, it can be stated that the adsorbed molecules form a monolayer on the surface of the adsorbent that is homogeneous (all the adsorption centers are identical) [63]. The Freundlich isotherm is used to characterize a multilayer adsorption process on a heterogeneous surface [63].

**Figure 19:**
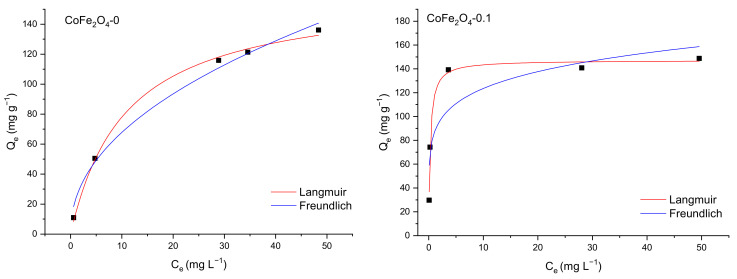

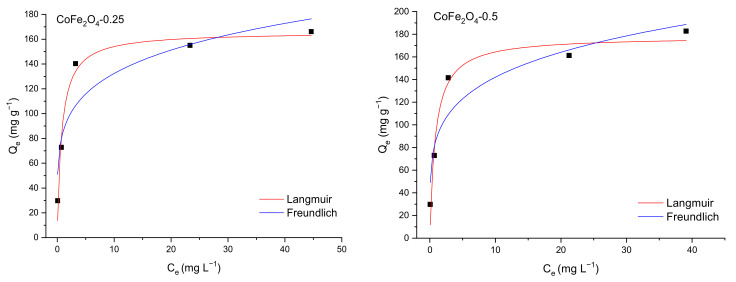
Langmuir and Freundlich fitting curves for CR adsorption onto CoFe_2_O_4_ samples.

**Figure 20:**
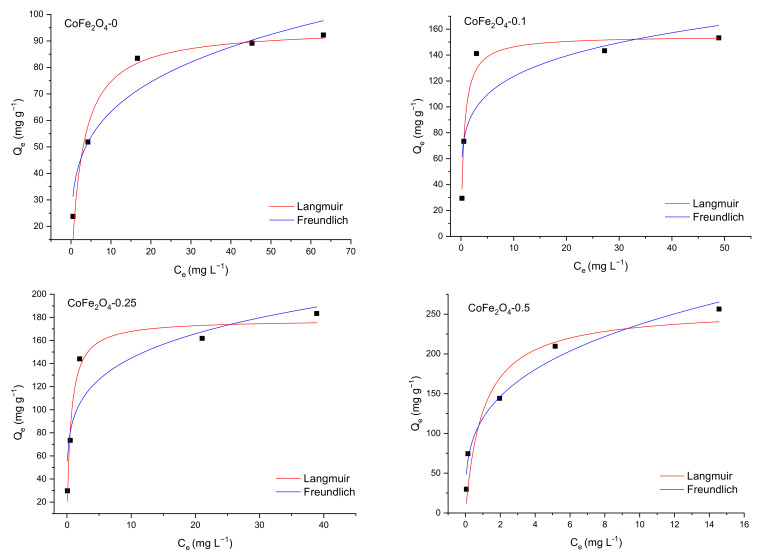
Langmuir and Freundlich fitting curves for MO adsorption onto CoFe_2_O_4_ samples.

In our study, the nonlinear forms of the isotherm equations were applied to characterize the dye adsorption process onto CoFe_2_O_4_ from single solutions. The curve fit of Langmuir and Freundlich isotherms for the two dyes in single and multicomponent solutions are shown in Figure 19 and Figure 20, while the parameters of both isotherms are listed in Table 7. Based on the values of the adjusted R^2^, it is obvious that the experimental data for the adsorption of the two dyes onto the CoFe_2_O_4_ samples is appropriately described by the Langmuir model, which implies that the adsorption process of CR and MO takes place on the homogeneous surface of the synthesized cobalt ferrite as a monolayer and each dye molecule does not interact with the neighboring one.

The maximum value of the adsorption capacity determined from the Langmuir isotherm varies from 162.15 to 178.07 mg g^−1^ for CR and from 94.99 to 257.25 mg g^−1^ for MO with the increase of the molar ratio CoFe_2_O_4_:DOSS from 1:0 to 1:0.5. The increase of the adsorption capacity is proportional with the increase of the molar ratio CoFe_2_O_4_:DOSS.

For binary solutions, the competitive adsorption capacity of dyes can be evaluated using the modified Langmuir isotherm model. This model is mathematically described by Equation (9) [64].

(9) $$Q_{e,\mathrm{dye}1}=\frac{Q_{\max,\mathrm{dye}1}K_{L,\mathrm{dye}1}C_{e,\mathrm{dye}1}}{1+K_{L,\mathrm{dye}1}C_{e,\mathrm{dye}1}+K_{L,\mathrm{dye}2}C_{e,\mathrm{dye}2}}$$

Equation (9) is transformed into Equation (10) by linearization:

(10) $$\frac{1}{Q_{e,\mathrm{dye}1}}=\frac{1}{Q_{\max,\mathrm{dye}1}}+\frac{1}{Q_{\max,\mathrm{dye}1}K_{L,\mathrm{dye}1}}\;\left[\frac{1}{C_{e,\mathrm{dye}1}}+\frac{K_{L,\mathrm{dye}2}C_{e,\mathrm{dye}2}}{C_{e,\mathrm{dye}1}}\right]\;$$

For the second dye from binary solutions, Equation (10) turns into Equation (11):

(11) $$\frac{1}{Q_{e,\mathrm{dye}2}}=\frac{1}{Q_{\max,\mathrm{dye}2}}+\frac{1}{Q_{\max,\mathrm{dye}2}K_{L,\mathrm{dye}2}}\;\left[\frac{1}{C_{e,\mathrm{dye}2}}+\frac{K_{L,\mathrm{dye}1}C_{e,\mathrm{dye}1}}{C_{e,\mathrm{dye}2}}\right]\;$$

where C_e_,_dye1_, C_e_,_dye2_, Q_e,dye1,_ and Q_e,dye2_ are the equilibrium concentration and the equilibrium adsorption capacity of dye 1 and dye 2 in the binary systems, K_L,dye1_ and K_L,dye2_ are Langmuir constants characteristics for the dye adsorption from single solutions, Q_max,dye1_ and Q_max,dye2_ are maximum adsorption capacities of dye 1 and dye 2 onto CoFe_2_O_4_ from the binary solution [54,64].

From the linear plot $\frac{1}{Q_{e,\mathrm{dye}1}}$ versus $\left[\frac{1}{C_{e,\mathrm{dye}1}}+\frac{K_{L,\mathrm{dye}2}C_{e,\mathrm{dye}2}}{C_{e,\mathrm{dye}1}}\right]$ it can be calculated Q_max,dye1_ in binary systems. For the second dye, Q_max,dye2_ can be obtained by the same method using Equation (11).

The ratio $\frac{Q_{\max,\mathrm{binary}}}{Q_{\max,\sin\mathrm{gle}}}$ provides information regarding the dynamics of dye adsorption in binary systems [65]. When this ratio is supraunitary, the two adsorbates have a synergistic effect, with the mixture’s effect stronger than the individual adsorbate’s effect. When the ratio is smaller than one, the two adsorbates have an antagonistic effect, with a weaker effect of the mixture than that of each of the individual adsorbates. When $\frac{Q_{\max,\mathrm{binary}}}{Q_{\max,\sin\mathrm{gle}}}=1$, the mixture has no effect on the adsorption of each of the dyes in the mixture [65]. Q_max,CR_ and Q_max,MO_ values, and the ratio $\frac{Q_{\max,\mathrm{binary}}}{Q_{\max,\sin\mathrm{gle}}}$ for each CoFe_2_O_4_ sample, calculated using the above-described algorithm, are shown in Table 8.

In the case of CR adsorption, the ratio $\frac{Q_{\max,\mathrm{binary}}}{Q_{\max,\mathrm{single}}}$ is smaller than 1 for all adsorbents, excepting CoFe_2_O_4_-0.25, suggesting that the presence of MO inhibits CR adsorption and the mixture has a weaker effect than the individual adsorbates in the mixture. The size of the dye molecules and the texture of the adsorbent both play a role in this phenomenon. Because CR molecules are substantially larger (2.3 nm) than MO molecules (1.2 nm) [66], it is very likely that the presence of MO molecules in the binary solution will limit their adsorption onto CoFe_2_O_4_ with a porous structure (pore widths between 2.5 and 4.5 nm). In the case of MO adsorption from binary solutions, for the adsorbents with the average pore size ≥ 3.5 nm (CoFe_2_O_4_-0 and CoFe_2_O_4_-0.1) the ratio $\frac{Q_{\max,\mathrm{binary}}}{Q_{\max,\mathrm{single}}}$ is supraunitary, suggesting a synergistic behavior of the two adsorbates, while for the other two adsorbents, CoFe_2_O_4_-0.25 and CoFe_2_O_4_-0.5, for which the average pore size is smaller than 3.5 nm, the two adsorbates have an antagonistic behavior.

The adsorption capacity of the CoFe_2_O_4_ samples for the removal of CR and MO was compared (Table 9) with those of other similar adsorbents reported in the literature. It is obvious that our samples show superior performance in terms of adsorption of the two dyes, compared to other similar materials.

Based on the above results, the adsorption behavior of CR or MO by CoFe_2_O_4_ can be mainly assigned to the following: (i) large specific surface area of CoFe_2_O_4_ samples that enhances the mass-transfer process; (ii) the electrostatic interactions between the positively-charged surface of the CoFe_2_O_4_ samples and the negative sulfonate groups of CR/MO; (iii) the H-bonds established between the OH groups of CoFe_2_O_4_ and NH_2_/SO^3-^/azo (-N=N-) groups of CR/MO. The differences between the adsorption capacities of the CoFe_2_O_4_ samples for these two dyes can be explained based on their molecular size [66].

#### 3.5.4. Desorption Study

**Figure 21:**
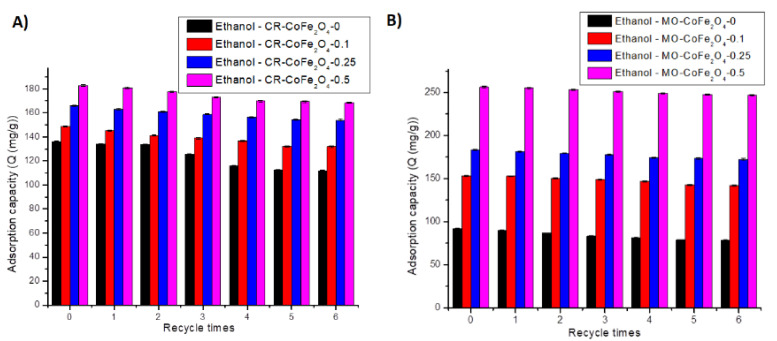
Reusability of CoFe_2_O_4_ samples for CR (**A**) and MO (**B**).

**Figure 22:**
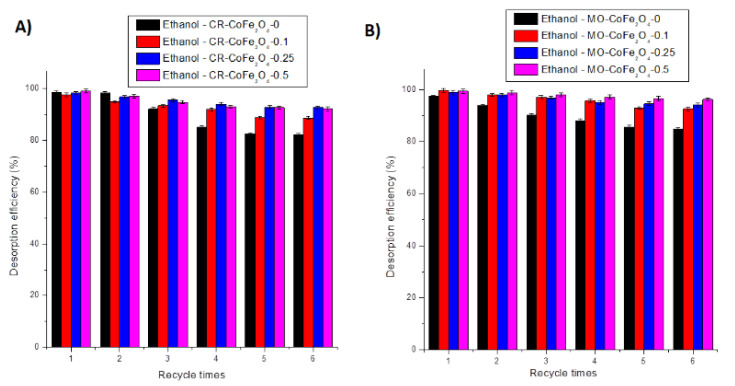
Desorption efficiency of CoFe_2_O_4_ samples for CR (**A**) and MO (**B**).

The regeneration capacity and the possibility to be applied in multiple adsorption-desorption systems are relevant adsorbent properties especially for industrial applications. Ethanol has been tested as a desorbing agent due to its high dipole moments [76]. Six adsorption-desorption cycles in ethanol were applied to determine the reusability of the prepared adsorbents. The results of the adsorption-desorption tests are provided in Figure 21A,B. Desorption efficiency has been determined as well and the results are presented in Figure 22A,B.

By analyzing the results presented in Figure 21 and Figure 22, it can be concluded that after six adsorption-desorption tests the maximum decrease of the adsorption capacity was 24.17 mg·g^−1^ for CR and 13.96 mg·g^−1^ for MO, respectively, for the CoFe_2_O_4_-0 sample. The minimum decrease of the adsorption capacity was noticed for the CoFe_2_O_4_-0.5 sample. The desorption efficiency of ethanol varied between 82.24 and 99.01% for CR and between 84.86 and 99.42% for MO, respectively. After six adsorption-desorption consecutive tests, the CoFe_2_O_4_ samples lost 15–18% of the desorption efficiency. This result demonstrates the possibility of using the CoFe_2_O_4_ samples for repetitive adsorption-desorption cycles.

## 4. Conclusions

In this study, the influence of sodium bis-2-ethyl-hexyl-sulfosuccinate (DOSS) surfactant on the properties of cobalt ferrite nanoparticles (size, shape, texture, and magnetic properties) and also on their ability to remove anionic dyes (Congo Red and Methyl Orange) from synthetic single and binary aqueous solutions was investigated. The obtained cobalt ferrite nanoparticles have high saturation magnetization (Ms) values ranging from 11.7 to 33.5 emu/g, indicating that they can be isolated quickly and efficiently from solution after adsorption using an external magnetic field. This represents a big advantage because it overcomes the limits of standard separation procedures such as sedimentation and filtration. The effect of different parameters such as contact time and solution pH on the adsorption process was systematically investigated. The results showed that a pH value of 4.5 is the most favorable for CR/MO adsorption. An increase in the adsorption capacity of the CoFe_2_O_4_ samples with the increasing of the quantity of surfactant used in synthesis was observed. The adsorption process was satisfactorily described using the pseudo-first-order kinetic model and the Langmuir isotherm model. The results of the intraparticle diffusion model indicated that the adsorption process is controlled by external mass transfer and intraparticle diffusion.

The maximum adsorption capacity values are higher for MO than for CR in single solutions. In binary solutions it was observed that the presence of MO inhibits CR adsorption, excepting the sample CoFe_2_O_4_-0.25, this behavior being attributed to the size of the dye molecules and the texture of the adsorbent. In the case of MO adsorption from binary solutions, for the adsorbents with the average pore size ≥ 3.5 nm (CoFe_2_O_4_-0 and CoFe_2_O_4_-0.1) the two dyes have a synergistic behavior, while for the other two adsorbents with an average pore size smaller than 3.5 nm (CoFe_2_O_4_-0.25 and CoFe_2_O_4_-0.5), the two dyes have an antagonistic behavior. Ethanol was favorably used as a desorbing agent. A small decrease of the adsorption capacity of the CoFe_2_O_4_ samples was observed after six adsorption-desorption cycles. The CoFe_2_O_4_ samples studied in this research can be considered promising materials for removing CR and MO dyes from aqueous solutions in a cost-effective and efficient manner.

## Figures and Tables

**Table 1:** Textural, magnetic properties, lattice parameters and mean size of CoFe_2_O_4_ samples.

Sample	S_BET_ (m^2^g^−1^)	Pore Volume (cm^3^g^−1^)	Average Pore Size (nm)	M_S_ (emu/g)	*a* (Å)	*D* (nm)
CoFe_2_O_4_–0	186.0	0.160	3.50	33.5	8.329	5
CoFe_2_O_4_–0.1	171.3	0.170	3.66	28.5	8.329	5
CoFe_2_O_4_–0.25	175.3	0.163	3.41	20.1	8.329	4
CoFe_2_O_4_–0.5	201.7	0.216	3.35	11.7	8.392	3

**Table 2:** The kinetic models used to characterize the CR/MO adsorption onto CoFe_2_O_4_ particles [54].

Kinetic Model	Nonlinear Form of the Kinetic Equation		Defining the Parameters of Mathematical Equations
Pseudo-first-order	$Q_{t}=Q_{e}(1-e^{-k_{1}t})$	(4)	k_1_—the pseudo-first-order rate constant (min^−1^), Qe—the adsorption capacity at equilibrium (mg·g^−1^), Q_t_—the amount of CR/MO adsorbed at time t (mg·g^−1^),
Pseudo-second-order	$Q_{t}=\frac{Q_{e}^{2}k_{2}t}{1+Q_{e}k_{2}t}$	(5)	k_2_—the rate constant of the pseudo-second-order adsorption process (g·mg^−1^ min^−1^), Qe—the adsorption capacity at equilibrium (mg·g^−1^), Q_t_—the amount of CR/MO adsorbed at time t (mg·g^−1^),
Intraparticle diffusion	$Q_{t\;}=\;k_{id}t^{0.5}\;+\;C$	(6)	k_id_—the intraparticle diffusion rate constant (mg·g^−1^⸳ min^−1^), C—the boundary-layer thickness (mg·g^−1^).

**Table 3:** The kinetic parameters for dyes adsorption onto CoFe_2_O_4_ samples from single component solutions (nonlinear regression).

**Sample**	**CoFe_2_O_4_-0**	**CoFe_2_O_4_-0.1**	**CoFe_2_O_4_-0.25**	**CoFe_2_O_4_-0.5**
		**CR**		
Q_e_ exp (mg·g^−1^)	130.51	148.72	166.10	182.71
*Pseudo-first-order model*				
k_1_ (min^−1^)	0.0160 ± 0.0009	0.0145 ± 0.0006	0.0131 ± 0.0007	0.0124 ± 0.0009
Q_e_ cal (mg·g^−1^)	131.73 ± 2.65	153.29 ± 2.24	173.94 ± 3.56	195.87 ± 5.54
R^2^_adjusted_	0.9918	0.9965	0.9945	0.9907
*Pseudo-second-order model*				
k_2_ (10^−4^ g·mg^−1^·min^−1^)	0.8610 ± 0.0910	0.6419 ± 0.0466	0.4869 ± 0.0413	0.4023 ± 0.0577
Q_e_ cal (mg·g^−1^)	170.50 ± 4.85	201.73 ± 4.08	232.87 ± 5.71	264.43 ± 11.17
R^2^_adjusted_	0.9935	0.9973	0.9967	0.9912
		**MO**		
Q_e_ exp (mg·g^−1^)	92.19	153.29	183.29	256.32
*Pseudo-first-order model*				
k_1_ (min^−1^)	0.0409 ± 0.0037	0.0243 ± 0.0023	0.0235 ± 0.0016	0.0166 ± 0.0015
Q_e_ cal (mg·g^−1^)	87.50 ± 2.00	147.44 ± 4.15	179.20 ± 3.57	259.83 ± 7.89
R^2^_adjusted_	0.9471	0.9604	0.9816	0.9776
*Pseudo-second-order model*				
k_2_ (10^−3^ g·mg^−1^·min^−1^)	0.5522 ± 0.0522	0.1560 ± 0.0146	0.1201 ± 0.0062	0.4885 ± 0.0067
Q_e_ cal (mg·g^−1^)	98.10 ± 1.71	175.32 ± 3.72	214.72 ± 2.59	329.58 ± 11.88
R^2^_adjusted_	0.9834	0.9900	0.9970	0.9869

**Table 4:** The kinetic parameters for dyes adsorption onto CoFe_2_O_4_ samples from binary solutions (nonlinear regression).

**Sample**	**CoFe_2_O_4_-0**	**CoFe_2_O_4_-0.1**	**CoFe_2_O_4_-0.25**	**CoFe_2_O_4_-0.5**
		**CR**		
Q_e_ exp (mg·g^−1^)	88.87	98.46	101.32	115.64
*Pseudo-first-order model*				
k_1_ (min^−1^)	0.0109 ± 0.0007	0.0108 ± 0.0074	0.0122 ± 0.0087	0.0103 ± 0.0015
Q_e_ cal (mg·g^−1^)	95.76 ± 2.56	105.66 ± 2.99	105.85 ± 3.56	127.23 ± 8.06
R^2^_adjusted_	0.9954	0.9948	0.9933	0.9769
*Pseudo-second-order model*				
k_2_ (10^−4^ g·mg^−1^·min^−1^)	0.6613 ± 0.0740	0.6098 ± 0.0116	0.7253 ± 0.0710	0.4636 ± 0.0084
Q_e_ cal (mg·g^−1^)	133.25 ± 4.62	146.60 ± 0.8644	143.24 ± 4.15	178.58 ± 102
R^2^_adjusted_	0.9966	0.9985	0.9968	0.9982
		**MO**		
Q_e_ exp (mg·g^−1^)	85.55	117.34	131.65	158.22
*Pseudo-first-order model*				
k_1_ (min^−1^)	0.0396 ± 0.0037	0.0252 ± 0.0019	0.0261 ± 0.0025	0.0212 ± 0.0026
Q_e_ cal (mg·g^−1^)	81.63 ± 1.94	113.02 ± 2.44	125.02 ± 3.45	154.17 ± 5.77
R^2^_adjusted_	0.9744	0.9848	0.9742	0.9617
*Pseudo-second-order model*				
k_2_ (10^−3^ g·mg^−1^·min^−1^)	0.5590 ± 0.0690	0.2130 ± 0.0119	0.2034 ± 0.0019	0.1256 ± 0.0208
Q_e_ cal (mg·g^−1^)	91.94 ± 2.13	133.91 ± 1.68	147.33 ± 3.07	185.88 ± 7.32
R^2^_adjusted_	0.9871	0.9977	0.9933	0.9814

**Table 5:** Kinetic parameters obtained from the fitting of the experimental data with the intraparticle diffusion model.

**Sample**	**Breakpoint (min^1/2^)**	**k_id_ (mg·g^−1^·min^−1/2^)**	**C (mg·g^−1^)**	**R^2^**
**RC Adsorption**				
CoFe_2_O_4_–0	7.3	15.09	−30.67	0.9890
	14.2	7.44	25.15	0.9968
CoFe_2_O_4_–0.1	7.8	15.15	−27.20	0.9978
	13.8	9.50	17.11	0.9906
CoFe_2_O_4_–0.25	7.4	15.32	−24.41	0.9875
	13.8	11.92	0.87	0.9991
CoFe_2_O_4–_0.5	6.7	−14.52	14.34	0.9949
	13.2	−2.18	15.48	0.9948
		**MO Adsorption**		
CoFe_2_O_4_–0	5.72	13.68	−10.70	0.9669
	14.49	2.79	51.64	0.9866
CoFe_2_O_4_–0.1	5.26	20.59	−26.21	0.9999
	13.94	8.16	39.08	0.9985
CoFe_2_O_4_–0.25	6.92	19.78	−15.73	0.9942
	13.70	9.15	57.92	0.9999
CoFe_2_O_4–_0.5	7.74	21.28	−9.68	0.9547
	13.31	17.94	16.13	0.9856

**Table 6:** The Langmuir and Freundlich nonlinear isotherms.

Isotherm Model	Nonlinear Form of the Isotherm Equation		Defining the Parameters of Mathematical Equations
Langmuir	$Q_{e}=\frac{Q_{\max}K_{L}C_{e}}{1+K_{L}C_{e}}$	(7)	C_e_ = equilibrium concentration of the solute in the solution (mg·L^−1^), K_L_ = the equilibrium constant of the Langmuir model related to the adsorption energy (L·mg^−1^), Q_e_ = the adsorption capacity at equilibrium (mg·g^−1^), Q_max_ = the maximum adsorption capacity (mg·g^−1^).
Freundlich	$Q_{e}=K_{F}\times C_{e}^{\frac{1}{n}}$	(8)	K_f_ and 1/n = Freundlich adsorption isotherm parameters (adsorption capacity (mg·g^−1^) and intensity), C_e_ = the equilibrium concentration of the solute in the solution (mg·L^−1^).

**Table 7:** Langmuir and Freundlich parameters for dye adsorption onto CoFe_2_O_4_ samples from single component solutions.

**Dye**	**CR**			
**Sample**	**CoFe_2_O_4_-0**	**CoFe_2_O_4_-0.1**	**CoFe_2_O_4_-0.25**	**CoFe_2_O_4_-0.5**
Langmuir Parameters				
Q_max_ (mg·g^−1^)	162.1564 ± 5.6842	147.2104 ± 3.7878	165.8074 ± 8.4989	178.0762 ± 10.3876
K_L_ (L·mg^−1^)	0.0927 ± 0.0118	3.7115 ± 0.5291	1.3116 ± 0.3681	1.1944 ± 0.3614
R^2^_adjusted_	0.9966	0.9866	0.9616	0.9546
Freundlich Parameters				
K_F_ (mg·g^−1^)	23.3615 ± 3.7796	86.1721 ± 15.1046	85.0676 ± 14.1775	88.0141 ± 13.3722
1/n	0.4629 ± 0.0454	0.1563 ± 0.0550	0.1920 ± 0.0538	0.2079 ± 0.0502
R^2^_adjusted_	0.9876	0.7372	0.8310	0.8741
**Dye**	**MO**			
**Sample**	**CoFe_2_O_4_-0**	**CoFe_2_O_4_-0.1**	**CoFe_2_O_4_-0.25**	**CoFe_2_O_4_-0.5**
Langmuir Parameters				
Q_max_ (mg·g^−1^)	94.9929 ± 5.1054	154.8871 ± 6.4913	178.0100 ± 8.5675	257.2528 ± 37.9185
K_L_ (L·mg^−1^)	0.3666 ± 0.1108	1.7089 ± 0.3616	1.6430 ± 0.4021	0.9819 ± 0.6989
R^2^_adjusted_	0.9533	0.9702	0.9674	0.8802
Freundlich Parameters				
K_F_ (mg·g^−1^)	36.8004 ± 6.1289	82.7225 ± 17.6008	91.8601 ± 15.7835	119.1167 ± 9.1690
1/n	0.2354 ± 0.0472	0.1740 ± 0.0678	0.1970 ± 0.0570	0.2988 ± 0.0351
R^2^_adjusted_	0.9107	0.6786	0.8150	0.9718

**Table 8:** Maximum adsorption capacity calculated using Langmuir and modified Langmuir isotherm models for single component and binary solutions.

Adsorbent	Dye	Parameters	Single Component Solution (mg·g^−1^)	Binary Solution (mg·g^−1^)	${\frac{Q_{\max,\mathrm{binary}}}{Q_{\max,\mathrm{single}}}}_{\;}$
CoFe_2_O_4_-0	CR	Q_max,CR_	163.89	118.06	0.72
	MO	Q_max,MO_	95.36	120.91	1.26
CoFe_2_O_4_-0.1	CR	Q_max,CR_	149.99	111.23	0.74
	MO	Q_max,MO_	161.09	170.94	1.06
CoFe_2_O_4_-0.25	CR	Q_max,CR_	170.40	214.59	1.25
	MO	Q_max,MO_	187.98	135.31	0.71
CoFe_2_O_4_-0.5	CR	Q_max,CR_	187.10	147.05	0.78
	MO	Q_max,MO_	262.03	116.27	0.44

**Table 9:** Adsorption capacities of different adsorbents with spinel structure from the literature for the removal of CR and MO.

Dye	Adsorbent	Adsorption Capacity (mg·g^−1^)	Reference
CR	Mo-doped CoFe_2_O_4_	135.14	[67]
	CoFe_2_O_4_	185.4	[68]
	MnFe_2_O_4_	92.4	[40]
	NiFe_2_O_4_	97.1	[40]
	Fe_3_O_4_	68.5	[40]
	ZnFe_2_O_4_ nanospheres	16.1	[69]
	Co_0.3_Ni_0.7_Fe_2_O_4_	131.75	[70]
	FeFe_2_O_4_	97.42	[71]
	CoFe_2_O_4_-0	162.15	This study
	CoFe_2_O_4_-0.1	147.21	This study
	CoFe_2_O_4_-0.25	165.80	This study
	CoFe_2_O_4_-0.5	178.07	This study
MO	CoFe_2_O_4_–FGS nanocomposites	71.54	[72]
	CuFe_2_O_4_@CeO_2_ nanofibers	100.0	[73]
	Co_3_O_4_ nanoparticles	46.08	[74]
	CoFe_2_O_4_	94.33	[75]
	ZnFe_2_O_4_	49.43	[75]
	Co_0.5_Zn_0.5_Fe_2_O_4_	67.1	[75]
	CoFe_2_O_4_-0	94.99	This study
	CoFe_2_O_4_-0.1	154.88	This study
	CoFe_2_O_4_-0.25	178.01	This study
	CoFe_2_O_4_-0.5	257.25	This study
